# Double-Sided Nano-ZnO: Superior Antibacterial Properties and Induced Hepatotoxicity in Zebrafish Embryos

**DOI:** 10.3390/toxics10030144

**Published:** 2022-03-18

**Authors:** Mingyue He, Xueting Li, Lidong Yu, Shuai Deng, Ning Gu, Li Li, Jianbo Jia, Bingsheng Li

**Affiliations:** 1 School of Life Science and Technology, Harbin Institute of Technology, Harbin 150080, China; hemingyue@hit.edu.cn (M.H.); 18644081918@163.com (X.L.); shuaideng@cuhk.edu.hk (S.D.); guning@hit.edu.cn (N.G.); 2 School of Physics, Harbin Institute of Technology, Harbin 150080, China; yulidong@stu.hit.edu.cn; 3 School of Biotechnology and Health Sciences, Wuyi University, Jiangmen 529020, China; 4 Key Laboratory of UV Light Emitting Materials and Technology of Ministry of Education, Northeast Normal University, Changchun 130024, China; libs@nenu.edu.cn

**Keywords:** Nano-ZnO, zebrafish, antibacterial, anti-inflammatory, oxidative stress, endoplasmic reticulum stress, hepatotoxicity, NAFLD, carbon dots

## Abstract

Zinc oxide nanoparticles (Nano-ZnO) have been widely used in the food, cosmetics, and biomedical fields due to their excellent antibacterial and antioxidant properties. However, with the widespread application of Nano-ZnO, Nano-ZnO inevitably enters the environment and living organisms, causing harm to human health and ecosystem safety. Therefore, the biosafety and toxicological issues of Nano-ZnO are gradually being emphasized. Our study found that Nano-ZnO has superior antibacterial properties compared to ofloxacin in the fight against *Staphylococcus aureus* (*S. aureus*). Given that ofloxacin can inhibit bacterial-induced inflammation, we constructed a model of bacterial inflammation using *S. aureus* in zebrafish. We found that Nano-ZnO inhibited the NF-κB-mediated inflammatory signaling pathway. However, in the process, we found that Nano-ZnO caused hepatic steatosis in zebrafish. This suggested that Nano-ZnO had a certain hepatotoxicity, but did not affect liver development. Subsequently, we investigated the mechanism of hepatotoxicity produced by Nano-ZnO. Nano-ZnO triggered oxidative stress in the liver by generating ROS, which then induced endoplasmic reticulum stress to occur. It further activated *srebp* and its downstream genes *fasn* and *acc1*, which promoted the accumulation of fatty acid synthesis and the development of steatosis, leading to the development of nonalcoholic fatty liver disease (NAFLD). To address the hepatotoxicity of Nano-ZnO, we added carbon dots for the treatment of NAFLD. The carbon dots were found to normalize the steatotic liver. This provided a new strategy to address the hepatotoxicity caused by Nano-ZnO. In this work, we systematically analyzed the antibacterial advantages of Nano-ZnO in vivo and in vitro, explored the mechanism of Nano-ZnO hepatotoxicity, and proposed a new method to treat Nano-ZnO hepatotoxicity.

## 1. Introduction

In recent years, Nano-ZnO has been found to exhibit many special functions in catalysis, optics, magnetism, and mechanics. It has become a multifunctional material that is widely used in cosmetics, medical supplies, biomedical and other daily biochemical products, such as optical devices [1], photocatalysts [2,3], automatic cleaning coatings [4], paints [5], textile industry [6], medical devices [7], dentistry [8], sunscreens [9], cosmetics [10], medical bioimaging, drug/gene delivery drug delivery [11,12], etc. Earlier studies on Nano-ZnO focused on the antimicrobial effect in vitro. It can be used as an effective antimicrobial agent in ointments, lotions, mouthwashes, and surface coatings of various medical devices to prevent microbial adhesion, colonization, and spread [13,14,15]. However, there has been no report on the antimicrobial activity of Nano-ZnO in vivo.

With the increasing release of Nano-ZnO-containing products into the environment, especially the aquatic environment, it is easy accumulation in aquatic organisms to occur, causing toxic effects. In addition, Nano-ZnO can easily enter the body through inhalation, ingestion, or skin barrier. It is concentrated in various organs and difficult to excrete [15]. Therefore, the biosafety and toxicological issues of Nano-ZnO should not be ignored. Nano-ZnO enters the body through different pathways such as respiratory, digestive and parenteral routes and accumulates mainly in the heart, liver, spleen, kidneys and lungs. Researchers believe that it produces oxidative stress and endoplasmic reticulum stress, causing liver damage [16]. It has been shown that Nano-ZnO induces apoptosis in mouse liver through oxidative stress [17]. Nano-ZnO also causes apoptosis in human hepatocytes (HepG2), lung epithelial cells and some cancer cells through the production of reactive oxygen species (ROS) [18,19,20]. These results confirm that oxidative stress triggered by ROS is one of the possible mechanisms of Nano-ZnO-induced toxicity.

Zebrafish (Danio rerio) has become a major model organism in the field of toxicology and drug screening because of its small size, easy management, high spawning capacity, transparent early embryos, and ease of observation [21]. Previous studies have shown that Nano-ZnO can inhibit the development and hatching of zebrafish embryos [22,23]. Acute Nano-ZnO exposure can induce the production of excessive ROS causing DNA damage [24]. It has also been found that Nano-ZnO inhibits development and other life activities by affecting the cell cycle in zebrafish [25]. However, there is no research to solve the toxicity of Nano-ZnO.

In this study, we conducted a systematic study from the antibacterial properties of Nano-ZnO to the anti-inflammatory response and its side effects. We explored the developmental toxicity and hepatotoxicity of Nano-ZnO and further studied the mechanism of liver injury caused by Nano-ZnO and how to mitigate the liver injury caused by Nano-ZnO. Our study found that the same concentration of Nano-ZnO was more effective antibacterial than ofloxacin. In addition, it inhibited the production of bacterial inflammation in zebrafish, but with some hepatotoxicity. Nano-ZnO has also been reported to be hepatotoxic [16]. To overcome this problem, we introduced egg-white-based carbon dots (EWCDs) with the function of treating iron-overloaded NAFLD prepared by our team [26]. We found that EWCDs could repair Nano-ZnO-induced liver damage and alleviate Nano-ZnO-induced hepatotoxicity. This provides guidelines for the future application of Nano-ZnO and post-application treatment.

## 2. Materials and Methods

### 2.1. Materials

Zebrafish is a wild-type strain AB, which was purchased from Heilongjiang Institute of Fisheries, Chinese Academy of Fisheries Sciences. Trizol, PrimeScriptTM RT reagent Kit with gDNA Eraser, Premix TaqTM and SYBR^®^ Premix Ex TaqTM II (Tli RNaseH Plus) were bought from Takara (Tokyo, Japan). PCR primers were synthesized by Jilin Kumei Biotechnology Co., Ltd, Cahngchun, China. Powder-form Nano-ZnO was obtained from Sigma Co., Ltd. (Product No. 677450, Berlin, Germany). In addition, the morphological data of Nano-ZnO were examined in our previously published article [27].

The automatic light-controlled circulating water system for zebrafish feeding was purchased from Taiwan Chuanfu Science and Technology Development Co., Ltd. (Taipei, China), the SPX-250BIII biochemical incubator of Tester was used for embryo culture. Sterilization operation used GI54DWS type autoclave. qRT-PCR was performed using Applied Biosystems ABI 7500. DNA and RNA purity analysis used Thermo Fisher NanoDrop 2000. Gel imaging system Tanon 1600 was purchased from Tanon Science & Technology Co., Ltd. (Shanghai, China). Other conventional equipment included metal bath, water bath, centrifuge, electronic balance, electrophoresis apparatus, etc.

### 2.2. Method

#### 2.2.1. Anti-Bacterial Properties of Nano-ZnO

Staphylococcus aureus cultured in triangular vials was coated on a plate. Cover slides coated with Nano-ZnO and ofloxacin were placed in the middle of the plate and incubated in a 37 °C for 24 h. Measurement of bacteriostatic zone diameter.

#### 2.2.2. Zebrafish Rearing and Mating

The AB strain zebrafish used in the experiment was raised in the (AAE-022-AA-A, Taiwan) circulating water system. The water temperature in the circulatory system is 28 °C, and the photoperiod is 14 L:10 D. To obtain healthy zebrafish embryos, adult fish with normal body shape and good physiological condition were selected for mating, and fish eggs with the same physiological cycle and normal development were collected. The collected fish eggs were washed with deionized water and then divided into control and treated groups and placed in a six-well plate for 20 pieces per well. In addition, they were kept in incubators with a constant temperature of 28 °C. Among them, the control group was cultured in E3 medium, and the test group was exposed to different concentrations of Nano-ZnO solution for subsequent gene expression studies.

#### 2.2.3. Establishment of Inflammation Model in Zebrafish

Adult zebrafish with normal body shape, agile predation, undamaged scales and fins and flexible swimming were selected as experimental objects. Zebrafish were cultured in sterile water for 24 h and randomly divided into 4 groups. After anesthesia with 0.3% tricaine, the muscle tissue of zebrafish tail was cut with scalpel in ultra-clean table. Sterile water was added to the scratches as control group (Control 2), 10^7^ CFU/mL Staphylococcus aureus suspension as bacterial inflammation group (*S. uresus*), 125 nmol/mL Nano-ZnO and 10^7^ CFU/mL bacterial suspension co-treatment group (Nano-ZnO). After 20 min, the zebrafish were put back into sterile water for feeding. After 24 h, the fish were compared with the uninjured control group (Control 1) for observation. Body fluids at the wounds of the zebrafish were extracted for plate coating, and the total number of bacteria on the plate was calculated after incubation at 37 °C for 24 h.

#### 2.2.4. Acute Toxicity Test of Nano-ZnO on Zebrafish

The concentration gradient of the Nano-ZnO in the experimental group was designed to be 0, 31.25, 62.5, 125, 250 nmol/mL, 4 mL of the above zinc oxide solution was added to the single well for exposure to the treatment group, and the control group was cultured with equal volume of E3 water. Zebrafish were kept in incubators with a constant temperature of 28 °C. A six-hole plate is a parallel, with three groups. The development of zebrafish embryos was observed at different time points after fertilization (24 h/48 h/72 h/96 h). The survival rate at four time points, the hatching rate of 72 h, the heart rate at 48 h, and the rate of malformation at 96 h were recorded, and the typical malformation occurred was counted and photographed (The research is supported by the Experimental Animal Welfare Ethics Committee of Harbin Institute of Technology. The ethical approval code is IACUC-2019022. Approval date: 6 December 2019).

#### 2.2.5. Detection of Zebrafish Cell Apoptosis

Apoptosis of zebrafish juveniles was detected by acridine orange staining. At 96 hpf, 10 zebrafish embryos from the control group and 31.25 nmol/mL Nano-ZnO treatment group were selected. After PBS washing, AO staining agent diluted with PBS and finally diluted at 2 mg/L was added for staining. After immersion in the dark for 20 min, the PBS was washed. The luminescence area of the control group and the treated group was observed under a fluorescence microscope to determine the effect of Nano-ZnO on the apoptosis of zebrafish embryo cells.

#### 2.2.6. Gene Expression Analysis

The zebrafish used in the experiment were tested for apoptosis gene, liver development gene and endoplasmic reticulum stress gene, and the relative expression of the gene was analyzed by semi-quantitative method. First, the total RNA of the sample material was extracted by Trizol method. The collected samples were lysed and homogenized by Trizol (Invitrogen, Sigma), and then passed through chloroform, isopropanol and 75% ethanol (both from Tianjin Zhiyuan Chemical Reagent Co., Ltd., Tianjin, China). DEPC water was purchased from Beijing Biotopped Science & Technology Co., Ltd. (Beijing, China). Centrifugal washing was performed to obtain the desired total RNA, and RNA was reverse-transcribed into cDNA as a PCR template using TaKaRa’s PrimeScript RT reagent Kit with gDNA Eraser kit. PCR reaction conditions: 95 °C for 5 min, 95 °C for 30 s, 72 °C for 30–60 s, 28–40 cycles, 2 °C for 4 min. PCR primers are shown in Table S3.

#### 2.2.7. Oil Red Staining

Juvenile zebrafish from different treatment groups were collected and placed in 4% paraformaldehyde solution overnight at 4 °C for fixation. The tissues were dehydrated with 40%, 60% and 80% of 1,2-propanediol solution, respectively. The dehydrated samples were then stained overnight in 0.5% oil red o solution. The stained zebrafish were eluted using 80%, 60%, and 40% 1,2-propanediol solutions, and the non-specifically stained fraction was removed and preserved by adding 80% glycerol. The fattened portions are stained orange when observed under the microscope.

#### 2.2.8. HE Staining, Picric Acid-Aspirate Scarlet Staining

Liver tissues from control and Nano-ZnO-treated groups were taken, fixed, dehydrated, transparent, wax-impregnated and embedded for paraffin embedding. The paraffin-embedded samples were cut into 4 μm slices. After dewaxing, hematoxylin eosin (H&E) staining or dropwise staining with saturated picric acid-aspirate scarlet stain was performed, photographed, and observed under a light microscope.

### 2.3. Statistical Analysis and Processing of Results

(1) Survival rate: The number of surviving embryos compared to the total number of embryos.
(2) Hatching rate: The number of hatching embryos compared to the total number of embryos.
(3) Heart rate: The number of embryonic heartbeats within 15 s was counted under the microscope.
(4) Autonomous exercise frequency: The number of spontaneous movements of the embryo within 60 s under the microscope.
(5) Gene expression level: Based on the expression level of the ef1α internal reference gene, the electrophoresis band diagram was subjected to gray scale analysis using ImageJ software.
(6) Statistical analysis of results: The experimental results are expressed as mean ± standard error, and each experiment was repeated three times. Statistical analysis was performed on the results of the control group and the experimental group by *t*-test. *p* < 0.01 was considered to be significantly different, and *p* < 0.05 was significant.

## 3. Results and Discussion

### 3.1. Anti-Bacterial Properties of Nano-ZnO In Vivo and In Vitro

Current studies on the biology of Nano-ZnO are mainly focused on the antibacterial properties. Studies have shown that Nano-ZnO has antibacterial effects on both foodborne pathogens *E. coli* and *Staphylococcus aureus*, among which it is more sensitive to *S. aureus* [28]. Ofloxacin is a broad-spectrum antibacterial fluoroquinolone drug. It is mainly used for acute and chronic infections caused by gram-negative bacteria. It is a common clinical antibacterial drug at present. We chose to use a well-known antibiotic to make a visual comparison of the antibacterial ability of Nano-ZnO.

**Figure 1:**
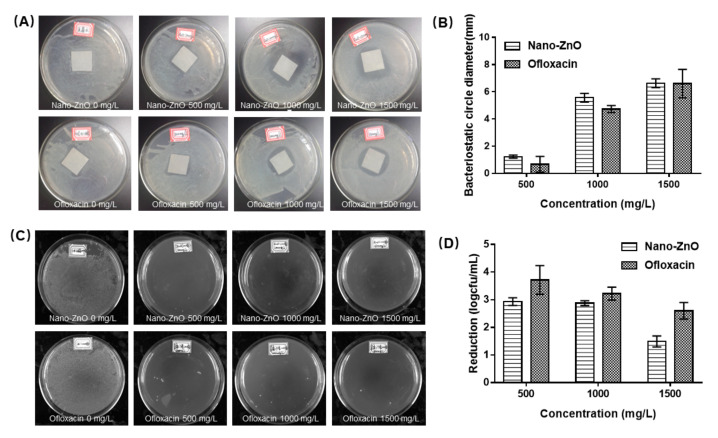
Antibacterial effect of Nano-ZnO on *S. aureus*: (**A**) inhibition circle of Nano-ZnO and ofloxacin, (**B**) quantitative column chart of (**A**), (**C**) single colony formation of Nano-ZnO and ofloxacin, (**D**) quantitative column chart of (**C**).

We used ofloxacin as a control to observe the antibacterial ability of Nano-ZnO. Versus against *S. aureus*. As the concentration of Nano-ZnO increased, its antibacterial effect against *S. aureus* was better, as shown in Figure 1. In vitro antibacterial assays demonstrated that Nano-ZnO was sensitive to *Staphylococcus aureus*. At the same mass concentration, Nano-ZnO and ofloxacin had similar antibacterial effects. However, in fact, with respect to the broad-spectrum antibacterial effect, ofloxacin was superior [29,30].

**Figure 2:**
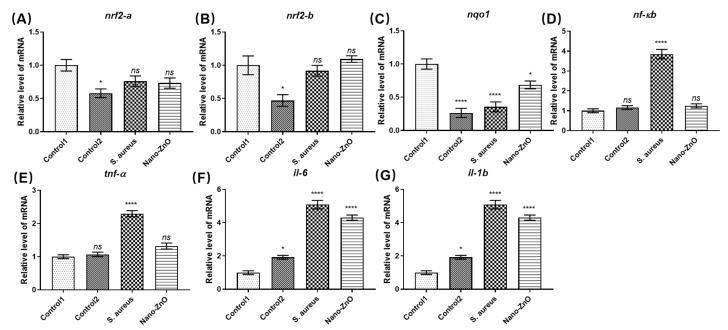
Effect of Nano-ZnO on the inflammatory response induced by *S. aureus*. Changes in mRNA levels of antioxidant signaling pathway (**A**) *nrf2-a*, (**B**) *nrf2-b*, (**C**) *nqo1*, and inflammatory signaling pathway (**D**) *nf-kb*, (**E**) *tnf-a*, (**F**) *il-6*, and (**G**) *il-1b*. (Control1: no treatment, Control2: wound + sterile water, *S. aureus*: wound + *S. aureus*, Nano-ZnO: wound + *S. aureus* + Nano-ZnO, *n* = 30, * *p* < 0.05, **** *p* < 0.0001).

We subsequently established a zebrafish bacterial inflammation model to study the in vivo anti-inflammatory effects of Nano-ZnO. Compared to control 1, we found that the antioxidant gene nqo1 was significantly decreased in the *S. aureus* group that underwent tail scratching, and its upstream Nrf2a, Nrf2b were not significantly changed (Figure 2A–C). The expression levels of inflammatory factors TNF-a, IL-1b, IL-6 and their upstream NF-κB were significantly increased (Figure 2D–G), indicating that scratching resulted in reduced antioxidant capacity and induced inflammation in zebrafish. Compared with the *S. aureus* group, Nano-ZnO enhanced the antioxidant capacity of wound-infected zebrafish and significantly reduced the inflammatory signaling pathways NF-κB and TNF-a. This indicated that Nano-ZnO had a certain inhibitory effect on the inflammation caused by *S. aureus*. The above results indicate that Nano-ZnO not only has excellent broad-spectrum antibacterial properties, but also inhibits the inflammatory response caused by bacteria. It can be used as a potential anti-infection drug as an alternative to ofloxacin.

### 3.2. Hepatotoxicity of Nano-ZnO in Zebrafish

**Figure 3:**
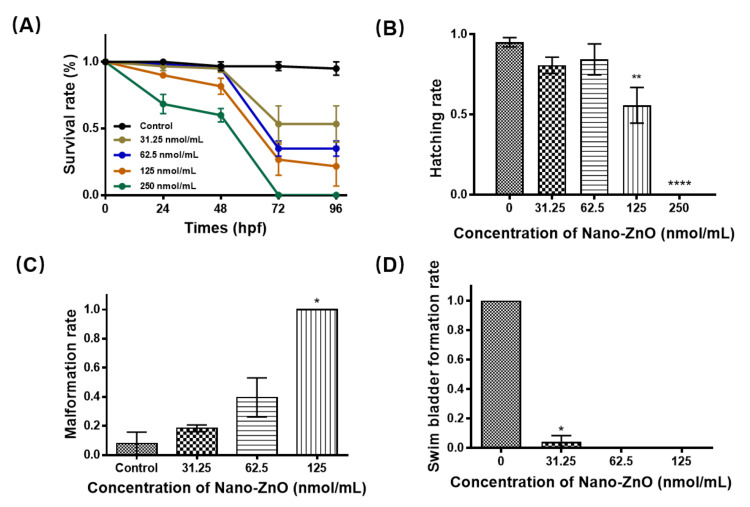
The effect of nano zinc oxide on (**A**) the survival rate, (**B**) the hatching rate, (**C**) the malformation rate, and (**D**) the swim bladder formation rate. Data are expressed as means ± SD. (*n* = 30, * *p* < 0.05, ** *p* < 0.01, **** *p* < 0.0001).

Although Nano-ZnO exhibited excellent resistance to infection, exposure to Nano-ZnO was toxic. We exposed zebrafish embryos to 0, 31.25, 62.5, 125, and 250 nmol/mL Nano-ZnO solutions, respectively. As shown in Figure 3A, the survival rate of zebrafish embryos was related to the concentration of Nano-ZnO solution. The survival rate of embryos decreased significantly as the concentration of Nano-ZnO solution increased. The semi-lethal concentration of Nano-ZnO was 31.25 nmol/mL. We remarked that between 48 hpf and 72 hpf, the survival rate of embryos decreased sharply. The embryos died without reaching the hatching stage, but the survival rate after hatching was essentially the same. This phenomenon suggested that Nano-ZnO caused zebrafish embryos to fail to hatch properly, resulting in death or delayed development. The hatching rate of embryos was calculated based on the number of surviving zebrafish embryos and the number of hatchings. The hatching rate of embryos showed a decreasing trend with the increase of Nano-ZnO concentration, as shown in Figure 3B. In the mortality and hatching rate statistics, we found that even though zebrafish survived, different types of malformations were present, and the malformation rate was shown in Figure 3C. Typical malformations in zebrafish embryos included incomplete yolk sac absorption, pericardial edema, curvature of the spine, and absence of the swim bladder. As shown in Figure 3D, most of these zebrafish had missing swim bladders. We counted and photographed the different types of malformations, as shown in Tables S1 and S2. As the concentration of the treatment solution increased, the number of surviving zebrafish embryos decreased, and the malformation rate gradually increased, indicating that Nano-ZnO was highly developmentally toxic.

**Figure 4:**
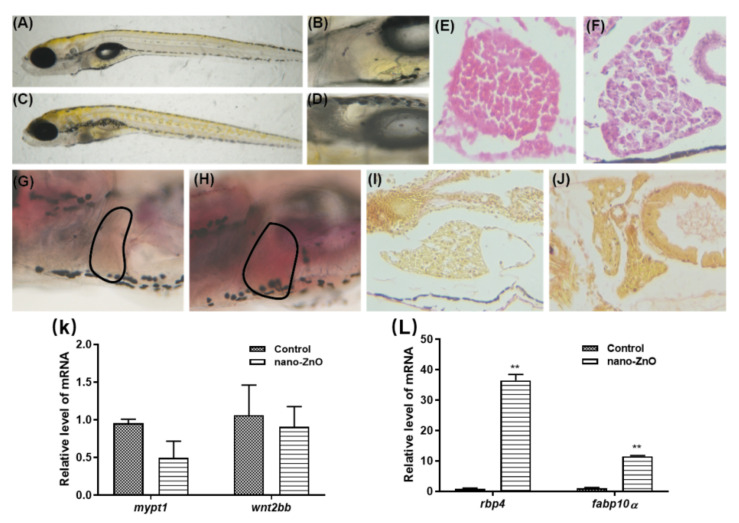
Hepatotoxic effects of 125 nmol/mL Nano-ZnO on zebrafish. Live observation of zebrafish liver differences between the control group and the Nano-ZnO treatment group: (**A**) control group (250×), (**B**) control group (410×), (**C**) Nano-ZnO treatment group (250×), (**D**) Nano-ZnO treatment group (410×). H&E staining of zebrafish liver: (**E**) control group (410×), (**F**) Nano-ZnO treatment group (410×). Oil red staining of zebrafish liver steatosis: (**G**) control group (410×), (**H**) Nano-ZnO treatment group (410×). Picric acid-Sirius scarlet staining of zebrafish liver steatosis: (**I**) control group (410×), (**J**) Nano-ZnO treatment group (410×). (**K**) Liver development-related genes relative level of mRNA. (**L**) Liver disease-related genes relative level of mRNA ** *p* < 0.01.

The malformation of the yolk sac is mainly characterized by hepatotoxicity. As the largest detoxification and metabolism organ, the liver can promote the metabolism of some toxic substances and then excrete them out of the body, thus playing a detoxifying role [31]. We treated zebrafish with Nano-ZnO at a semi-lethal concentration of 31.25 nmol/mL. The differences in liver development and morphology between the experimental and control groups were compared. We saw the deepened color and larger size of the liver in the experimental group, as shown in Figure 4C. When the liver was observed under magnification, it could be seen that the liver of the control zebrafish was pale yellow with a relatively free shape and indistinct outline, as shown in Figure 4B. In contrast, the liver of zebrafish in the experimental group was well-defined and black in color with a good “vacuum”-like structure, as shown in Figure 4D. These changes suggested that Nano-ZnO might have some effects on the liver of zebrafish. Furthermore, the microstructural changes of the liver were observed by H&E staining, as shown in Figure 4E,F. The hepatocytes were neatly and closely arranged in the control group. However, in the experimental group, the spacing between cells was large, the structure of the liver was loose, and the vacuolous structure inside the cells might be fat. In addition, then, we used oil red to stain the liver of zebrafish. Our experiments revealed that the liver of zebrafish in the experimental group were stained light red, while the control group remained light yellow, as shown in Figure 4G,H. These results indicate that Nano-ZnO has a certain hepatotoxicity to zebrafish. We found that zebrafish liver staining was similar in the experimental and control groups, with the same fiber type and the same degree of fibrosis by picric acid-Sirius scarlet staining, as shown in Figure 4I,J. This indicated that the Nano-ZnO-induced steatosis in zebrafish had not yet entered the fibrosis stage.

To further explore whether Nano-ZnO is toxic to liver development and to test whether the steatosis is due to underdevelopment of the liver. We examined the expression level of some liver development-related and typical liver lesion marker genes, as shown in Figure 4K,L. wnt2bb and mypt1 play important roles in the formation phase of liver progenitor cells. They are essential molecules for liver development in zebrafish [32]. It was found that the expression level of wnt2bb and mypt1 decreased after exposure to Nano-ZnO, but there was no significant difference. Meanwhile, there was little difference in the size and shape of the liver between the experimental and control groups and the degree of liver development was consistent. This indicated that Nano-ZnO had no effect on the development of the liver, as shown in Figure 4K. Retinol-binding protein (rbp4) is a member of the retinol-binding protein family and is mainly expressed in the liver and fat. When nonalcoholic fatty liver disease (NAFLD) occurs, the expression level of rbp4 is higher than normal [33]. In addition, fabp10 encodes the fatty acid binding protein (fabp), which is mainly expressed in the liver and small intestine. It has been reported that when NAFLD occurs, fabp expression is significantly higher than that in the control group [34]. As seen in Figure 4L, the expression of rbp4 and fabp10 genes was significantly higher in the experimental group than in the control group. This further confirmed the results of the preliminary oil red staining and H&E staining, and identified the Nano-ZnO-induced hepatotoxicity as nonalcoholic fatty liver.

### 3.3. Molecular Mechanism of Nano-ZnO Toxicity in Zebrafish

To further explain the toxic effects of Nano-ZnO on zebrafish liver, we explored the causes of liver damage caused by Nano-ZnO at the molecular level. Xia et al. suggest that the toxicity of Nano-ZnO originates from the leaching of zinc ions. This process generates ROS that destabilize zinc ions, inhibit enzymatic activity, and ultimately lead to cell death [35]. Another part of the study focuses on the dissolved oxygen produced by Nano-ZnO, which causes some oxidative damage to the organism [36,37]. In addition, zinc ion dissolution leads to cell membrane breakdown in normal cells, which further induces cells apoptosis [35].

**Figure 5:**
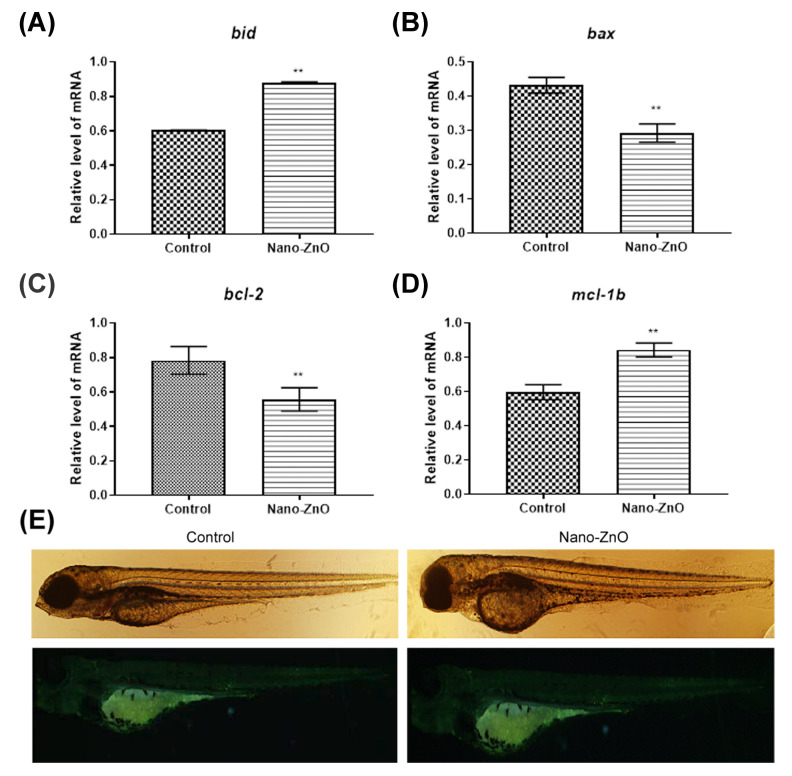
Effect of 125 nmol/mL Nano-ZnO on apoptosis in zebrafish. Changes in mRNA level of pro-apoptosis genes (**A**) bid and (**B**) bax, and anti-apoptosis genes (**C**) bcl-2 and (**D**) mcl-1b. (**E**) Acridine orange apoptosis staining. (*n* = 30, ** *p* < 0.01).

Our previous experiments revealed that there was a large amount of death in zebrafish embryos before hatching after high concentration of Nano-ZnO treatment. We hypothesized that apoptosis might be induced by zinc ions solubilization. Therefore, we examined the expression level of apoptotic genes and stained the apoptotic cells with acridine orange. The mitochondrial apoptotic pathway is regulated by proteins of the Bcl-2 family, which plays an anti-apoptotic or pro-apoptotic role. It is the predominant regulator of the mitochondrial apoptotic pathway. Bax and bid in the Bcl-2 family are pro-apoptotic, while bcl-2 and mcl-1b, in contrast, have the effect of inhibiting apoptosis and promoting cell survival [38]. The effect of Nano-ZnO on the expression of apoptotic genes is shown in Figure 5A–D. Compared with the control group, the pro-apoptotic gene bid was significantly up-regulated and the other pro-apoptotic gene bax was significantly down-regulated in the experimental group. Among the anti-apoptotic genes, mcl-1b was significantly up-regulated in the experimental group, showing an enhanced anti-apoptotic effect, while bcl-2 was somewhat down-regulated. bcl-2 and bax are opposing pairs of genes. If the trends are the same, the ratio of the two will be more meaningful. Our study showed that the ratio of bcl-2/bax was almost unchanged after Nano-ZnO exposure. Bid interacts with mcl-1. The trends of were both consistent. This suggested that Nano-ZnO exposure did not cause apoptosis. In addition, staining of apoptotic cells by acridine orange revealed that no significant apoptosis occurred in the experimental group, as shown in Figure 5E. This was consistent with our previous apoptosis gene assay results. The above results suggested that the mechanism of organism damage or even embryonic death caused by Nano-ZnO was not due to apoptosis caused by the breakdown of cell membrane by zinc ion solubilization.

**Figure 6:**
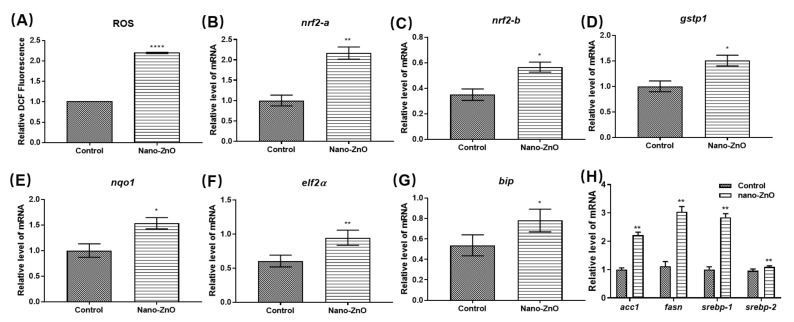
Mechanism of liver injury caused by 125 nmol/mL Nano-ZnO. (**A**) Relative levels of ROS; changes in mRNA levels of antioxidant signaling pathways (**B**) nrf2-a, (**C**) nrf2-b, (**D**) gstp1, and (**E**) nqo1; changes in mRNA levels of ER stress-related genes (**F**) elf2a and (**G**) bip. (**H**) Changes in mRNA levels of the lipid metabolism-related genes acc1, fasn, strebp-1 and strebp-2. (*n* = 30, * *p* < 0.05, ** *p* < 0.01, **** *p* < 0.0001).

Another cause of Nano-ZnO-induced toxicity is the generation of oxidative damage. This is caused by the ROS generated by the dissolution of zinc ions. We found that Nano-ZnO increased the level of ROS. Nano-ZnO induced oxidative stress in the organism, as shown in Figure 6A. Nano-ZnO stimulation caused oxidative stress in zebrafish embryos. This resulted in increased expression of the nrf2 gene, which further led to upregulation of the gstp1 gene as well as the nqo1 gene, enabling resistance to damage caused by oxidative stress, as shown in Figure 6B–E. Nano-ZnO disrupted the balance between oxidants and antioxidants in zebrafish, but this organismal defense system did not alleviate the damage caused by Nano-ZnO to the organism.

It has been shown that oxidative stress and endoplasmic reticulum stress (ER stress) are subject to crosstalk, and oxidative stress can activate ER stress [39]. To further investigate whether oxidative stress response triggers the generation of ER stress, we tested the expression of ER stress-related genes in zebrafish. Compared with the control group, the bands of ER stress-related genes were significantly brighter treated with Nano-ZnO in zebrafish. The ER stress genes bip and eif2a were significantly upregulated as shown in Figure 6F,G, indicating that Nano-ZnO induced ER stress. Several studies have shown that some important endoplasmic reticulum stress signaling pathways can intervene and regulate lipid metabolism in the liver through different mechanisms. Most importantly, it affects lipid metabolism by regulating the activation of srebp, which increases the synthesis of fatty acids in the liver. To further explore the mechanism of Nano-ZnO causing NAFLD, we examined the expression of lipid metabolism-related genes acc1, fasn, srebp-1 and srebp-2 after Nano-ZnO exposure. We found that Nano-ZnO promoted the expression of lipid synthesis genes. srebp-1 expression was promoted, which led to the expression upregulation of the downstream lipid synthesis genes fasn and acc1, as shown in Figure 6F. These changes activated the synthesis of fatty acids and increased their uptake by the liver. As a result, large amounts of lipids were deposited in the liver. Ultimately, these results led to lipid changes in the liver of zebrafish.

**Figure 7:**
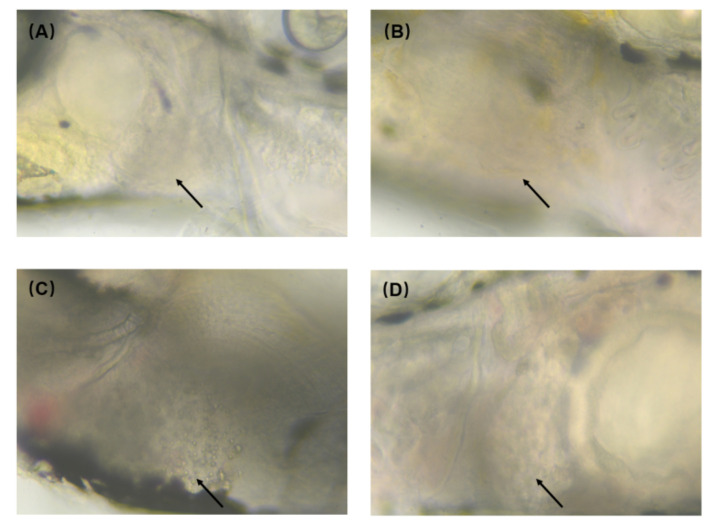
Alleviation of 125 nmol/mL Nano-ZnO toxicity by CDs: (**A**) control group, (**B**) CDs group, (**C**) Nano-ZnO group, (**D**) CDs + Nano-ZnO group. The arrow sites are zebrafish liver. (**A**,**B**,**D**) are normal zebrafish liver. (**C**) has obvious lipid droplets.

Zebrafish resist oxidative stimulation by Nano-ZnO through oxidative stress; however, the antioxidant Nrf2 signaling pathway does not completely eliminate ROS-induced liver injury. To address the NAFLD produced by Nano-ZnO exposure, we used pre-synthesized carbon dots to mitigate the liver injury caused by Nano-ZnO. The carbon dots were used to treat NAFLD. The CDs were extremely safe and did not cause liver damage compared to the control group, as shown in Figure 7B. However, after Nano-ZnO exposure, we found significant lipid droplet accumulation at the liver, as shown in Figure 7C. Surprisingly, EWCDs were found to effectively alleviate the accumulation of lipid droplets caused by Nano-ZnO, as shown in Figure 7D. The above results indicated that CDs could alleviate the liver damage caused by Nano-ZnO, which provided a new approach to solve the toxicity problem of Nano-ZnO.

## 4. Conclusions

In this work, we first explored the in vitro antibacterial effect of Nano-ZnO. It was found that Nano-ZnO has good antibacterial effect and can effectively inhibit the inflammatory response caused by *S. aureus*. However, Nano-ZnO showed a certain hepatotoxicity, mainly in the form of NAFLD. Secondly, we explored the mechanism of Nano-ZnO toxicity based on the current reports. We verified both apoptosis induced by zinc ion solubilization and ROS generated by zinc ion and found that Nano-ZnO does not cause apoptosis. We found that Nano-ZnO leads to oxidative stress mainly by promoting the production of ROS. Oxidative stress activates endoplasmic reticulum stress, which directly regulates the activation of genes related to fat metabolism in the srebp-fas pathway. This caused excessive uptake and formation of fatty acids, which were eventually deposited in the liver to form NAFLD. Nano-ZnO agglomerates when it enters the aqueous phase. Due to the agglomeration effect, the hydrodynamic diameter of Nano-ZnO in the solution is larger than its original particle size. It has been suggested that the toxicity of Nano-ZnO in aqueous environment depends on its hydrodynamic diameter, and within a certain range, the larger the hydrodynamic diameter, the more toxic it is [40]. The smaller-sized nanoparticles are more prone to agglomeration due to their larger surface energy [41,42]. Therefore, the smaller diameter of Nano-ZnO is more toxic. The average diameter of Nano-ZnO particles selected for this experiment was 35.65 ± 6.63 nm, and the small size may be another reason for the toxicity of Nano-ZnO. In addition, we introduced CDs for the first time to alleviate the hepatotoxicity of Nano-ZnO, which provided a new strategy to solve the toxicity of Nano-ZnO.

## Data Availability

Not applicable.

## Supplementary Materials

The following are available online at https://www.mdpi.com/article/10.3390/toxics10030144/s1. Table S1. Statistics of zebrafish malformations treated with different concentrations of nano-ZnO. Table S2. Development of zebrafish swim bladder treated with different concentrations of nano-ZnO. Table S3. Oligonucleotide primers.
