# Supplementary material for: Double-Sided Nano-ZnO: Superior Antibacterial Properties and Induced Hepatotoxicity in Zebrafish Embryos
Source: Toxics. 2022 Mar 18;10(3):144. doi: 10.3390/toxics10030144 (PMC8950655; doi:10.3390/toxics10030144)
Supplement: Supplementary file 1 [file toxics-10-00144-s001.zip › toxics-1617212-supplementary.pdf]

toxics MDPI

Supplementary Materials: Double-Sided Nano-ZnO: Superior Antibacterial Properties and Induced Hepatotoxicity in Zebrafish Embryos

Mingyue He, Xueting Li, Lidong Yu, Shuai Deng, Ning Gu, Li Li, Jianbo Jia and Bingsheng Li

Table S1. Statistics of zebrafish malformations treated with different concentrations of nano-ZnO.

Deformed parts	control	31.25 nmol/mL	62.5 nmol/mL	125 nmol/mL
**Yolk sac**	4/57	6/32	8/21	4/4
**Heart**	0/57	0/32	0/21	3/4
**Deformity rate**	4/57	6/32	8/21	4/4

Table S2. Development of zebrafish swim bladder treated with different concentrations of nano-ZnO.

Concentration	control	31.25 nmol/mL	62.5 nmol/mL	125 nmol/mL
**Normal development rate**	57/57	2/32	0/21	0/4

Table S3. Oligonucleotide primers.

Genes	Primer Sequences	Genes	Primer Sequences
ef1α-f	CCTGGGAGTGAAACAGCTGATC	*bid-f*	GTCCACCAGCGACCTACA
ef1α-r	CCGATCTTCTTGATGTATGCGCTG	*bid-r*	GCACCACTGTCCCATAAA
bax-f	GGAGGCGATACGGGCAGTG	*bcl-2-f*	TCGTTCAGACCCTCATTT
bax-r	TTGCGAATCACCAATGCTGTG	*bcl-2-r*	CATCCCAACCTCCATTTT
il-1b-f	TGGACTTCGCAGCACAAAATG	*srebp-1-f*	CATCCACATGGCTCTGAGTG
il-1b-r	GTTCACTTCACGCTCTTGGATG	*srebp-1-r*	CTCATCCACAAAAGAAGCGGT
tnf-α-f	GCTGGATCTTCAAAGTCGGGTGTA	*fabp10a-f*	GTGGGAGAATCGGTCAG
tnf-α-f	TGTGAGTCTCAGCACACTTCCATC	*fabp10a-r*	TCAGGAGAACTACGAGGAG
nf-κb-f	AAGATGAGAACGGAGACACGC	*rbp4-f*	AGGTGCCGTCCTCATCC
nf-κb-r	TACCAGCAATCGCAAACAACG	*rbp4-r*	CAACTTCGCCGTCCAAC
bip-f	AGCTGTACAAGAAGAAGAC	*chop-f*	GGACACGTAGAGAAGGGGAC
bip -r	GAAAGTTACCTCGATCTGTGGG	*chop-r*	TCTCGGTGGGAGACATTCAC
gstp1-f	CGACTGAAAGCCACCTGTGTC	*perk-f*	GACGAATGCTCCACTGTCCT
gstp1-r	CTGTCGTTTTTGCCATATGCAGC	*perk-r*	GTGTTGGACTTGACGATGCG
nqo1-f	TTTGCAGAATCCCGAGCACT	*bip-f*	AAGAGGCCGAAGAGAAGGAC
nqo1-r	TCTTCTGCGATCAAGCTGAAAG	*bip-r*	AGCAGCAGAGCCTCGAAATA
nrf2a-f	TGGACGAACACTAACACGGG	*mcl-1b-f*	CCAGAACTGAAAGCGCATA
nrf2a-r	CCTGCTGCATTGCGAAAGTT	*mcl-1b -r*	ACTCCACAAAGCCATCCC
nrf2b-f	GGCAGAGGGAGGAGGAGACCAT	*wnt2bb-f*	TATTGGAGCCCTTGGTGC
nrf2b-r	AAACAGCAGGGCAGACAACAAGG	*wnt2bb-r*	GCGGTGATGACGAAACTGA
eif2α-f	GGACAGATGGAAGTTTGGGATG	*mypt1-f*	AGCCTGCTGTCCCGGTATTG
eif2α-r	CGGTTTGTTGCGTAGAGCC	*mypt1-r*	GAGTGAGGTACGATCTGCG
